# Supplementary material for: Ex Vivo Study of Colon Health, Contractility and Innervation in Male and Female Rats after Regular Exposure to Instant Cascara Beverage
Source: Foods. 2024 Aug 6;13(16):2474. doi: 10.3390/foods13162474 (PMC11353626; doi:10.3390/foods13162474)
Supplement: Supplementary file 1 [file foods-13-02474-s001.zip › foods-3117616-supplementary.pdf]

foods

Supplementary Material

Ex Vivo Study of Colon Health, Contractility and Innervation in Male and Female Rats after Regular Exposure to Instant Cascara Beverage

Paula Gallego-Barceló [1,2 †], David Benítez-Álvarez [1,2,3,4 †], Ana Bagues [1,2,5,6*], Blanca Silván-Ros [1,2,7], Alba Montalbán-Rodríguez [2], Laura López-Gómez [1,2] Gema Vera [1,2,6], María Dolores del Castillo [2,8], José A. Uranga [1,2] and Raquel Abalo [1,2,6,9,10,*]

[1] Department of Basic Health Sciences, Faculty of Health Sciences, University Rey Juan Carlos (URJC), 28922 Alcorcón, Spain; paula.gallego@urjc.es (P.G.-B.); ana.bagues@urjc.es (A.B.); blanca.silvan@urjc.es (B.S.); alba.montalban@urjc.es (A.M-R); laura.lopez.gomez@urjc.es (L.L.-G.); gema.vera@urjc.es (G.V); jose.uranga@urjc.es (J.A.U.); raquel.abalo@urjc.es (R.A.)
[2] High Performance Research Group in Physiopathology and Pharmacology of the Digestive System (NeuGut-URJC), URJC, 28922 Alcorcón, Spain
[3] Institute of Human Genetics, Faculty of Medicine and University Hospital Cologne, University of Cologne, Kerpener Str. 34, 50931 Cologne, Germany; david.benitez-alvarez@uk-koeln.de (D.B.-A.)
[4] Center for Molecular Medicine Cologne (CMMC), Faculty of Medicine and University Hospital Cologne, University of Cologne, Robert-Koch-Str. 21, 50931 Cologne, Germany.
[5] High Performance Research Group in Experimental Pharmacology (PHARMAKOM-URJC), URJC, 28922 Alcorcón, Spain
[6] Associated I+D+i Unit to the Institute of Medicinal Chemistry (IQM), Scientific Research Superior Council (CSIC), 28006 Madrid, Spain
[7] Department of Biochemistry, Medical University of Lodz, 92-215 Lodz, Poland
[8] Food Bioscience Group, Department of Bioactivity and Food Analysis, Institute of Food Science Research (CIAL) (CSIC-UAM), Nicolás Cabrera Street, 9, 28049 Madrid, Spain; mdolores.delcastillo@csic.es (M.D.d.C.)
[9] Working Group of Basic Sciences on Pain and Analgesia of the Spanish Pain Society, 28046 Madrid, Spain.
[10] Working Group of Basic Sciences on Cannabinoids of the Spanish Pain Society, 28046 Madrid, Spain.
* Correspondence: ana.bagues@urjc.es (A.B.) Tel.: +34-91-488-86-65; raquel.abalo@urjc.es (R.A.) Tel.: +34-91-488-88-54
† These authors contributed equally to this work.

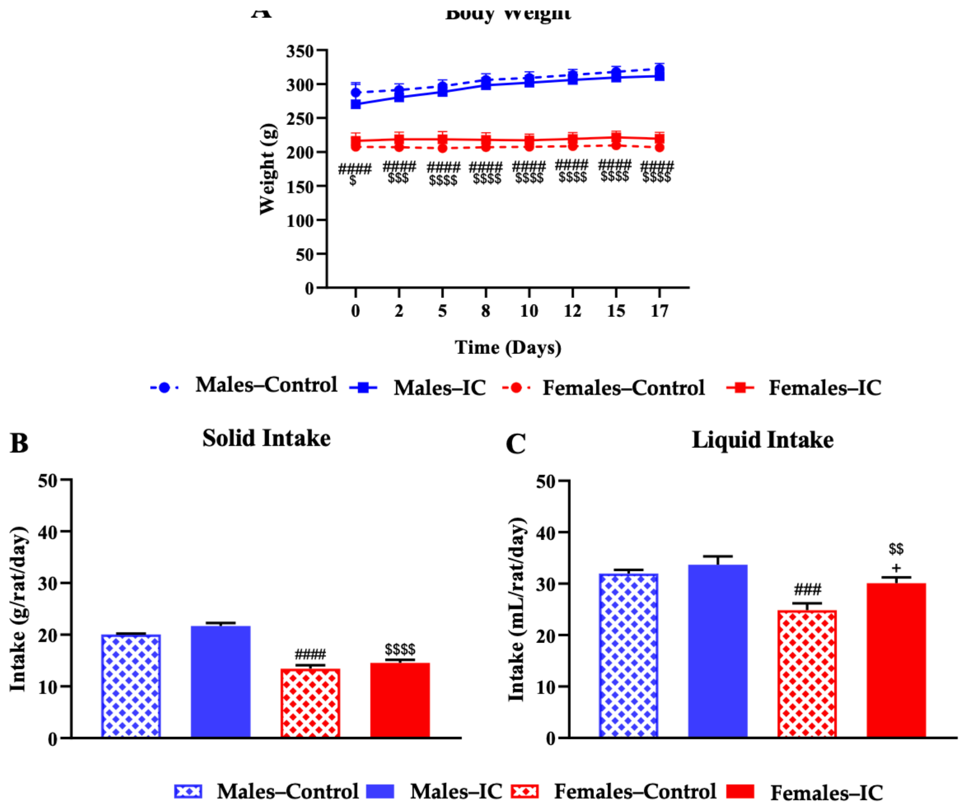

Figure S1. Evolution of body weight and solid and liquid intake in rats of both sexes exposed to Instant Cascara (IC) beverage. Evolution of body weight (A) and the overall mean solid (B) and liquid (C) intakes by each experimental group are shown. These parameters were recorded in the four experimental groups, distributed according to sex and the administered beverage (IC or water): Males–Control, Males–IC, Females–Control and Females–C. Data represent mean ± SEM (standard error of the mean). N = 6 animals per group. Sex-dependent statistically significant changes: ### $p<0.001$, #### $p<0.0001$ [Females–Control vs Males–Control]; $ $p<0.05$, $$ $p<0.01$, $$$ $p<0.001$, $$$$ $p<0.0001$ [Females–IC vs Males–IC]. Beverage-dependent statistically significant changes: + $p<0.05$ [Females–IC vs Females–Control]. A: Two-way ANOVA followed by Bonferroni's post-hoc test. B, C: One-way ANOVA followed Bonferroni's post-hoc test.

Body weight, and food and liquid intakes of the 6 rats/group used in the *in vitro* study are shown in Figure S1. During the three weeks of the study, the male and female control groups exhibited a normal and expected body weight progression for their age (2–3 months old). The consumption of IC did not result in significant differences in the body weight evolution of male and female rats. Regarding intakes, both male rat groups (control and IC) showed higher solid and liquid intakes than female groups. Exposure to IC only showed a slight but significant increase ($p<0.05$) in liquid intake among females.

Table S1. Vaginal smears in female rats before sacrifice.

	Metestrus	Diestrus	Proestrus	Estrus
Females–Control	16.67%	0%	50%	33.33%
Females–IC	16.66%	50%	16,67%	16.67%

At the end of the study, just before sacrifice, vaginal smears in female rats were conducted. Two experimental groups (n=5–6/group), distributed according to the administered beverage (IC or water) were used: Females-Control and Females-IC. Data represent mean ± SEM. Differences were not statistically significant among groups (p>0.05). Chi square test.

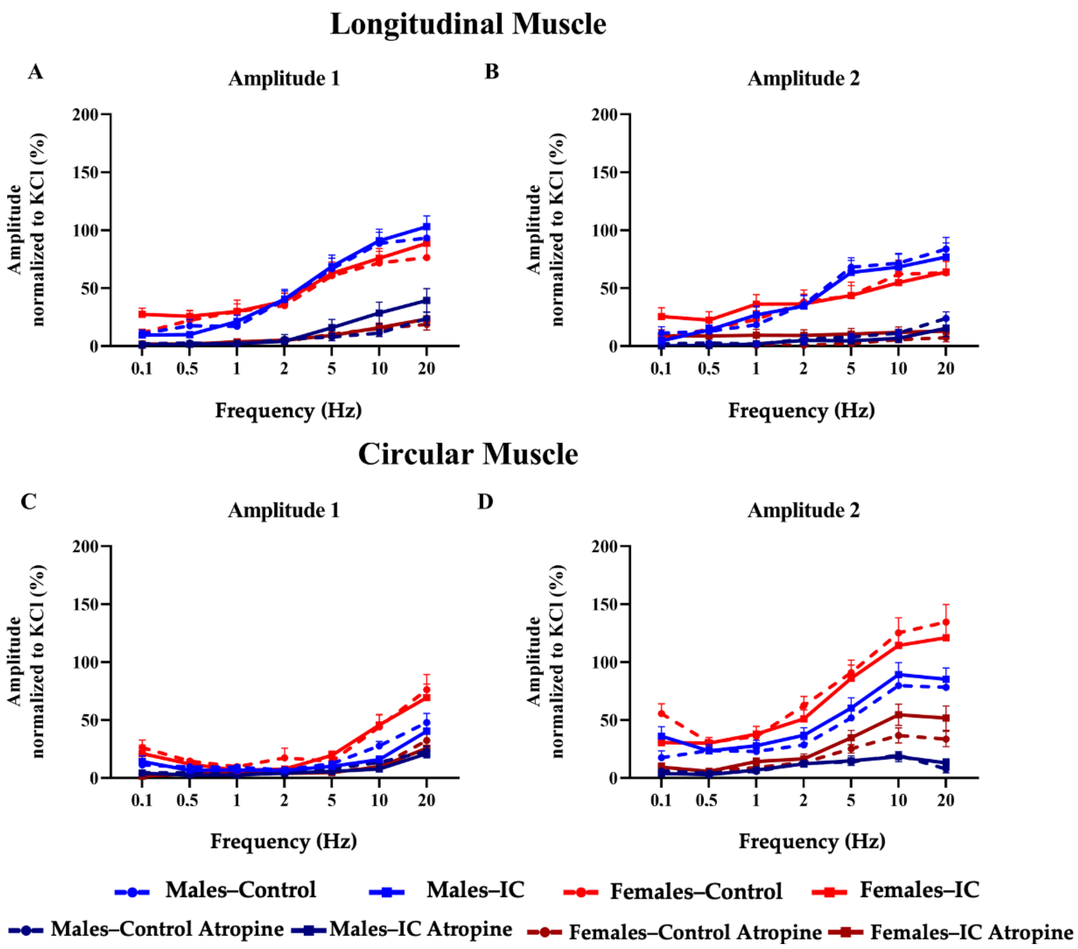

Figure S2. Longitudinal (LM) and Circular (CM) muscle response to electrical field stimulation (EFS). Muscle strips were electrically stimulated, in absence and presence of atropine (10^{-6} M), with 10-second trains of pulses (0.3 ms, 100 V) at frequencies of 0.1, 0.5, 1, 2, 5, 10 and 20 Hz. A: Maximum amplitude recorded during the 10 seconds of stimulation in LM (Amplitude 1); B: Maximum amplitude reached after stimulation (Amplitude 2) in LM; C: Amplitude 1 in CM; D: Amplitude 2 in CM. Data represent the mean ± SEM for each experimental group. N=5–6 rats/group; n=17–20 strips/group for both LM and CM. See Tables S1A–D show the results of the statistical analysis.

Table S2. Longitudinal muscle (LM) and Circular muscle (CM) response to electrical field stimulation (EFS).

EFS Frequency (Hz)				0.1	0.5	1	2	5	10	20
Experimental group (%)			Atropine							
Longitudinal muscle (LM)	Amplitude (A1)	Males–Control	–	10.8±2.9	17.5±5.6	17 ±4.7	40±7.8	66.7±9.5	88.6±10	93.2±10.6
			+	1.9±0.9	2.7±1.2	1.8±0.7	5.9±4.3$^{\circ\circ}$	7.9±3.1$^{\circ\circ\circ}$	11.3±3.2$^{\circ\circ\circ}$	24±5.8$^{\circ\circ\circ}$
		Males–IC	–	9.6±4.4	9.9±2.4	21.3±5.5	40.6±8.6	68.8±10.1	91±10.3	103±9.7
			+	0.9±0.6	1.6±0.7	2.3±0.8	4±1.3$^{\circ\circ\circ}$	15.9±7.4$^{\circ\circ\circ}$	28.6±9.6$^{\circ\circ\circ}$	39.4±10.5$^{\circ\circ\circ}$
		Females–Control	–	11.5±3.2	22.3±8.9	30±10.1	34.9±11.2	60.7±12.2	71.7±13	76.4±13.4
			+	0.5±0.4	1.8±0.6	3.3±1	4±0.9°	10.2±3.7$^{\circ\circ\circ}$	15.3±4.8$^{\circ\circ\circ}$	18.7±5$^{\circ\circ\circ}$
		Females–IC	–	27.3±5.6	25.7±4.9	29.9±6.8	38.8±6.8	62.8±7	75.7±6.3	88.8±6.1
			+	1.8±0.6	1.7±0.6	3.8±1.1$^{\circ\circ\circ}$	5.1±1.2$^{\circ\circ}$	9.2±1.4$^{\circ\circ\circ}$	15.8±2$^{\circ\circ\circ}$	23.4±2.7$^{\circ\circ\circ}$
	Amplitude (A2)	Males–Control	–	11.3±3.3	9.6±2	9.2±1.4	7.1±1.3	12.5±1.6	27.7±4.1	47.9±8.1
			+	4.6±1.7	4.5±1	3.1±0.7	3.8±1	8.3±2.6	13.4±4.5	23.1±4.4$^{\circ\circ}$
		Males–IC	–	14.2±5.2	6.6±1	6.9±1.2	6.7±1.4	10.1±1.6	16±3.1	40.4±6.4
			+	4.3±0.7	2.6±0.4	2.6±0.6	4.3±1.4	5.8±1.3	7.7±1.6	21.1±3.8°
		Females–Control	–	26.3±6.8	14.6±1.6	9.7±2.1	17.5±8.5	14.4±3	44.1±10.5	76.2±13.3
			+	3±1.1$^{\circ\circ}$	5.1±1.2	4.5±1.1	5.1±1.2	5.1±0.8	9.9±2$^{\circ\circ\circ}$	32.4±5.6$^{\circ\circ\circ}$
		Females–IC	–	21.1±7.4	12.1±2.2	6.6±1.7	7.8±1.2	19.6±3.8	45.7±9.5	69.5±12
			+	1.6±0.6°	3.8±1	3.9±0.8	4.1±0.9	4.7±1.3	9.7±2.6$^{\circ\circ\circ}$	25.8±5.4$^{\circ\circ\circ}$
Circular muscle (CM)	Amplitude (A1)	Males–Control	–	11.2±5.8	12.8±4.6	18.4±7.1	35.9±8	68.1±8.3	71.6±8.7	83.6±10.3
			+	1.9±0.9	2.7±1.2	1.8±0.7	5.9±4.3°	7.9±3.1$^{\circ\circ\circ}$	11.3±3.2$^{\circ\circ\circ}$	24±5.8$^{\circ\circ\circ}$
		Males–IC	–	4.7±3.1	13.9±4.4	27±7.8	34.6±9.3	63.5±10.7	68.3±11.3	76.9±12.6
			+	0.2±0.2	0.6±0.6	1.9±1.6	4.9±4.3°	4.7±2.3$^{\circ\circ\circ}$	6.8±3.6$^{\circ\circ\circ}$	15.4±7.3$^{\circ\circ\circ}$
		Females–Control	–	6±3.1	14.8±5.3	22.8±8	38.2±10.6	43.5±12.1	62±12.5	63.2±12.1
			+	0.7±0.8	0.4±0.5	0.6±0.6	0.8±0.6$^{\circ\circ}$	2.2±1.3$^{\circ\circ}$	5.7±2.7$^{\circ\circ\circ}$	7.3±3.7$^{\circ\circ\circ}$
		Females–IC	–	27.1±8.1	23.7±7.9	36.8±8.9	38.4±8.9	46.1±8.8	57.6±9.9	67.4±9.1
			+	8.9±5.4	8.8±5.1	9.4±5.1	9.3±4.9°	10.4±4.9$^{\circ\circ}$	11.9±4.8$^{\circ\circ\circ}$	12.9±4.4$^{\circ\circ\circ}$
	Amplitude (A2)	Males–Control	–	17.7±5.9	23.8±5.2	22.8±3.8	28.6±4.7	52±6.7	79.7±9.8	78.3±8.4
			+	6.1±2.3	4.5±1.6	5.7±1.7	12.9±2.7	13.9±3.1$^{\circ\circ}$	19.7±2.9$^{\circ\circ\circ}$	7.7±3.3$^{\circ\circ\circ}$
		Males–IC	–	36.1±8.4	23.5±3.7	28±4.7	36.9±6.8	60.4±9	89.2±10.7	85.3±9.9
			+	4±1.5°	2.8±0.9	6.9±2.5	12.3±3	14.9±4.2$^{\circ\circ\circ}$	18.4±4.4$^{\circ\circ\circ}$	13.1±3.6$^{\circ\circ\circ}$
		Females–Control	–	55.6±8.7	30.9±4.2	36 ±3.5	62.6±8	90.9±11.2	125.3±13.3	134.6±15.6
			+	6.4±2.9$^{\circ\circ\circ}$	6.7±2.3	10.2±2.5	14.4±2.8$^{\circ\circ\circ}$	27±3.7$^{\circ\circ\circ}$	39±7.2$^{\circ\circ\circ}$	35.6±7.4$^{\circ\circ\circ}$
		Females–IC	–	30.6±7.1	30.1±5	38±6.8	51±8.1	86.1±11.6	114.4±12.1	121.1±13.1
			+	9.4±3.6	5.8±2.5	14.3±3.1	16.6±4.2$^{\circ\circ\circ}$	34.8±6.5$^{\circ\circ\circ}$	54.6±9.4$^{\circ\circ\circ}$	51.7±10.8$^{\circ\circ\circ}$
